# Applying DEKOIS 2.0 in structure-based virtual screening to probe the impact of preparation procedures and score normalization

**DOI:** 10.1186/s13321-015-0074-6

**Published:** 2015-05-20

**Authors:** Tamer M Ibrahim, Matthias R Bauer, Frank M Boeckler

**Affiliations:** Laboratory for Molecular Design and Pharmaceutical Biophysics, Department of Pharmaceutical and Medicinal Chemistry, Institute of Pharmacy, Eberhard Karls University Tuebingen, Auf der Morgenstelle 8, 72076 Tuebingen, Germany; Department of Pharmaceutical Chemistry, Faculty of Pharmacy and Biotechnology, German University in Cairo, Cairo, 11835 Egypt

## Abstract

**Background:**

Structure-based virtual screening techniques can help to identify new lead structures and complement other screening approaches in drug discovery. Prior to docking, the data (protein crystal structures and ligands) should be prepared with great attention to molecular and chemical details.

**Results:**

Using a subset of 18 diverse targets from the recently introduced DEKOIS 2.0 benchmark set library, we found differences in the virtual screening performance of two popular docking tools (GOLD and Glide) when employing two different commercial packages (e.g. MOE and Maestro) for preparing input data. We systematically investigated the possible factors that can be responsible for the found differences in selected sets. For the Angiotensin-I-converting enzyme dataset, preparation of the bioactive molecules clearly exerted the highest influence on VS performance compared to preparation of the decoys or the target structure. The major contributing factors were different protonation states, molecular flexibility, and differences in the input conformation (particularly for cyclic moieties) of bioactives. In addition, score normalization strategies eliminated the biased docking scores shown by GOLD (ChemPLP) for the larger bioactives and produced a better performance. Generalizing these normalization strategies on the 18 DEKOIS 2.0 sets, improved the performances for the majority of GOLD (ChemPLP) docking, while it showed detrimental performances for the majority of Glide (SP) docking.

**Conclusions:**

In conclusion, we exemplify herein possible issues particularly during the preparation stage of molecular data and demonstrate to which extent these issues can cause perturbations in the virtual screening performance. We provide insights into what problems can occur and should be avoided, when generating benchmarks to characterize the virtual screening performance. Particularly, careful selection of an appropriate molecular preparation setup for the bioactive set and the use of score normalization for docking with GOLD (ChemPLP) appear to have a great importance for the screening performance. For virtual screening campaigns, we recommend to invest time and effort into including alternative preparation workflows into the generation of the master library, even at the cost of including multiple representations of each molecule.

**Graphical Abstract:**
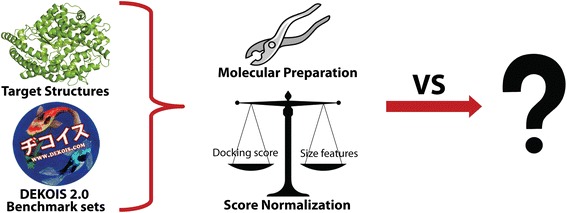
Using DEKOIS 2.0 benchmark sets in structure-based virtual screening to probe the impact of molecular preparation and score normalization.

**Electronic supplementary material:**

The online version of this article (doi:10.1186/s13321-015-0074-6) contains supplementary material, which is available to authorized users.

## Background

Virtual screening (VS) is a widely applied method in drug discovery that is used to predict novel bioactives from large chemical libraries [1–4]. In the last decade a multitude of successful VS applications have been reported [5–9]. In early-stage drug discovery especially structure-based virtual screening (SBVS) has been frequently used to identify new hits [10–14]. SBVS requires structures of target binding sites to predict potential interactions with ligand molecules. One of the most frequently used SBVS methods is molecular docking, which is used for docking molecules into the binding pocket to predict and score energetically favorable ligand binding modes [15–20].

Generally, it was found that VS performance depends strongly on the respective target properties [1, 21–24]. Different scoring and docking functions consequently favor different binding situations. To avoid wasting time and efforts on ineffective VS strategies, it is important to assess the performance of different VS setups in order to select the most effective workflow. Screening performance can be assessed using molecular benchmark sets, which consist of a set of bioactives and a set of inactive molecules, also known as decoys [23–25]. The higher the number of bioactives at the top of the score-ordered list of screened molecules, the better is the respective screening performance. Apart from the selection of a suitable docking tool, the success rate of VS tools depends strongly on various factors, such as the protonation/tautomerization state of the respective protein binding site residues [26] and of the input molecules [27–30], as well as the force field-minimized input conformation of the respective input molecules [31–35].

In this study, we investigate why and to which extent different protein/ligand preparation procedures affect VS performance by using our recently introduced DEKOIS 2.0 benchmark sets [21, 24, 36]. We test the widely applied docking tools GOLD and Glide with two comparable settings of input preparation using two different commercial packages (MOE and Maestro). In addition, we conduct a systematic in-depth analysis for one example (ACE, Angiotensin-I-converting enzyme) to evaluate the possible factors affecting the differences in screening performance between MOE and Maestro preparations. Interestingly, we found that the preparation of the bioactives caused a larger screening performance difference than the preparation of the decoys set or the target structure. Score normalization strategies eliminated the bias toward larger molecules in GOLD docking scores (ChemPLP) for ACE and also produced better performances in most of the other datasets.

## Results and discussion

### Selection of benchmark sets

To probe the impact of diverse docking setups on VS performance, suitable and diverse test datasets should be employed. Our recently introduced DEKOIS 2.0 library offers a wide variety of curated high-quality benchmark sets and is therefore well-suited for compiling a selection of evaluation kits [21, 24]. We selected 18 DEKOIS 2.0 datasets, each representing a different target class. Our compilation comprises proteases, kinases, transferases, oxido-reductases, nuclear receptors, and hydrolases, and describes various protein-ligand binding situations. This allows for a comprehensive analysis of performance differences for various VS setups. A complete list of selected datasets and the PDB codes of their target structures can be found in the Supporting Information (Additional file 1: Table S1).

### Ligand and protein preparation protocols

We aim at comparing the impact of two comparable preparation setups on VS performance employing two different preparation packages. Two of the most widely used applications for input preparation are MOE (Chemical Computing Group) and Maestro (Schrodinger). The preparation of the data was done at comparable levels of complexity for both programs, which are described in more detail in the Methods section. We conducted the preparation of the dataset (bioactives and decoys sets) by the LigPrep module in Maestro and the Wash and Minimize functions of MOE. Similarly, target structures were prepared by the ProtAssign function in Maestro and the Prot3D function of MOE.

### Impact of different preparation procedures on VS performance

We utilized the widely used pROC-AUC as a metric for the screening performance [21, 22, 24]. The pROC curve is a semi-logarithmic version of the standard linear curve of the receiver operating characteristics (ROC) [37]. Therefore, pROC curves are emphasizing the recognition of the early recovered hits when calculating the “area under the curve” (AUC) [37].

Table 1 shows the impact of different preparation schemes on docking performances (pROC-AUC) for diverse protein targets. Based on these findings, we defined the ΔpROC-AUC_*prep*_ as:

$$ \Delta \mathrm{pROC}\hbox{-} \mathrm{A}\mathrm{U}{\mathrm{C}}_{prep}=\mathrm{pROC}\hbox{-} \mathrm{A}\mathrm{U}\mathrm{C}\left(\mathrm{M}\mathrm{O}\mathrm{E}\right)\hbox{-} \mathrm{pROC}\hbox{-} \mathrm{A}\mathrm{U}\mathrm{C}\left(\mathrm{M}\mathrm{aestro}\right) $$

**Table 1:** Comparison of docking performances between MOE and Maestro data preparation schemes for diverse protein targets

Target	Glide			GOLD		
	Original pROC-AUC			Original pROC-AUC		
	MOE	Maestro	ΔpROC-AUC_*prep*_^*a*^	MOE	Maestro	ΔpROC-AUC_*prep*_
ACE	1.73	2.01	*−0.28* ^*b*^	1.55	1.25	***0.30***
ACHE	0.71	0.75	−0.04	0.72	0.72	0.00
ADRB2	0.97	0.89	*0.09*	0.74	0.65	*0.08*
CATL	0.72	0.81	*−0.09*	0.78	0.75	0.04
DHFR	1.12	0.63	***0.49*** ^*c*^	0.75	0.80	−0.05
ERBB2	2.01	1.80	*0.21*	1.85	2.12	*−0.27*
HDAC2	1.74	1.27	*0.47*	1.02	1.20	*−0.18*
HIVPR	1.54	1.40	*0.13*	1.46	1.64	*−0.18*
HSP90	0.74	0.61	*0.14*	0.42	0.33	*0.10*
JAK3	1.32	1.13	*0.19*	0.78	0.91	*−0.14*
JNK2	0.66	0.70	−0.04	0.77	0.74	0.03
MDM2	0.84	0.80	0.04	0.51	0.44	*0.07*
P38	1.79	1.48	*0.31*	0.49	0.56	*−0.07*
PI3KG	1.14	1.13	0.01	1.01	0.95	*0.06*
PNP	1.08	1.41	*−0.33*	0.98	1.04	*−0.07*
PPARg	0.90	1.11	*−0.20*	0.85	0.92	*−0.07*
Thrombin	2.00	1.93	0.07	1.22	1.15	*0.08*
TS	1.22	1.48	*−0.26*	1.27	1.05	*0.22*

^*a*^ΔpROC-AUC_*prep*_ is calculated as pROC-AUC (MOE) – pROC-AUC (Maestro). ^*b*^Italicized values represent significant deviations (ΔpROC-AUC_*prep*_), while the non-italicized values are deemed non-significant. ^*c*^Bold values represent the maximum ΔpROC-AUC_*prep*_ observed (for GOLD: ACE; for Glide: DHFR)

Because of the heuristic and the stochastic nature of the docking process in GOLD [31], we defined a “safety margin” of ±0.05 resulted from the deviation of multiple runs representing a non-significant change of ΔpROC-AUC_*prep*_, [24], and to avoid over-interpreting small changes in docking performance.

We observed that VS performances of GOLD for four of the 18 targets did not significantly differ between the two preparation schemes (Table 1). The remaining 14 targets showed larger changes. Seven of these targets (50 %) showed higher pROC-AUC values for Maestro prepared data, while the other 7 targets (50 %) benefitted from MOE data preparation. This suggests that the GOLD screening performance does not generally depend on a particular preparation scheme. For some examples the docking performance was affected dramatically by the different preparation schemes. ACE, ERBB2 and TS docking results showed relatively large ΔpROC-AUC_*prep*_ values, as shown in Table 1.

Despite the fact that the docking process in Glide is deterministic and multiple docking runs of the same input data yields exactly the same output every time [31], we still considered the “safety margin” of ±0.05 ΔpROC-AUC_*prep*_ to avoid over-interpreting small changes in docking performance. Four of the 18 targets did not significantly differ between the two preparations. Five of 14 targets (~36 %) showed higher pROC-AUC values for Maestro prepared data. For the remaining nine targets (~64 %), Glide produced higher VS performance with MOE preparation. The screening performance differed substantially between the preparation schemes for DHFR, HDAC2 and PNP (Table 1), while the remaining targets showed only smaller performance differences.

Such screening performance differences can be caused by various factors, such as (a) the selected protonation/tautomerization state of the respective protein binding site residues [26], (b) the protonation/tautomerization states of the respective input molecules of the dataset [27–29], and (c) the force field-minimized input conformation of the respective input molecules of the dataset [31–35]. To elucidate the influence of (a) and (b), we compared protonation/tautomerization states of the binding sites and the molecules and quantified the differences. To get an idea to which extent (c) differs, we calculated the pairwise RMSD of the conformers of the bioactives produced by the two preparations. A comprehensive summary of these results can be found in the Supporting Information (Additional file 1: Table S2 and Table S3).

In general, we observed that the screening performance differences between the two preparations are variable in a target-dependent manner for both docking programs. We observed that the metal-containing targets (e.g. ACE and HDAC2) showed relatively high ΔpROC-AUC_*prep*_ for both GOLD and Glide. This is not surprising, since the complexity of the metal-containing microenvironment of the binding sites is a well-known phenomenon [29, 38]. The targets that contained higher numbers of different protonation/tautomerization states (Additional file 1: Table S2 and Table S3) between the two preparations (particularly for the bioactives) frequently show higher ΔpROC-AUC_*prep*_ for both docking programs (e.g. ERBB2 and TS). When ignoring the special cases ACE and HIVPR, there is a certain trend for the remaining 16 datasets, suggesting that difference in protonation/tautomerization states of the bioactives and ΔpROC-AUC_*prep*_ are correlated (Additional file 1: Figure S1). This implies that the occurrence of different bioactive protomers and tautomers can be a major contribution to the obscured screening performance. Interestingly, in several cases (e.g. DHFR and HDAC2) where the ΔpROC-AUC_*prep*_ values between Glide and GOLD differ significantly, a higher number of different protonation/tautomerization states was found for the bioactives. For a more in-depth analysis, we focus on examining ACE in more detail.

### Impact of target, bioactive, and decoy preparation

We have chosen ACE, as a particular interesting example, because for this target we observed the highest ΔpROC-AUC_*prep*_ value between MOE and Maestro prepared data for GOLD and a large ΔpROC-AUC_*prep*_ for Glide. Therefore, we explored more thoroughly the various factors influencing the performance difference of GOLD. We also conducted a similar analysis on DHFR that showed the highest ΔpROC-AUC_*prep*_ for Glide (see Additional file 1: Figure S2 and Figure S3 in SI).

**Fig. 1:**
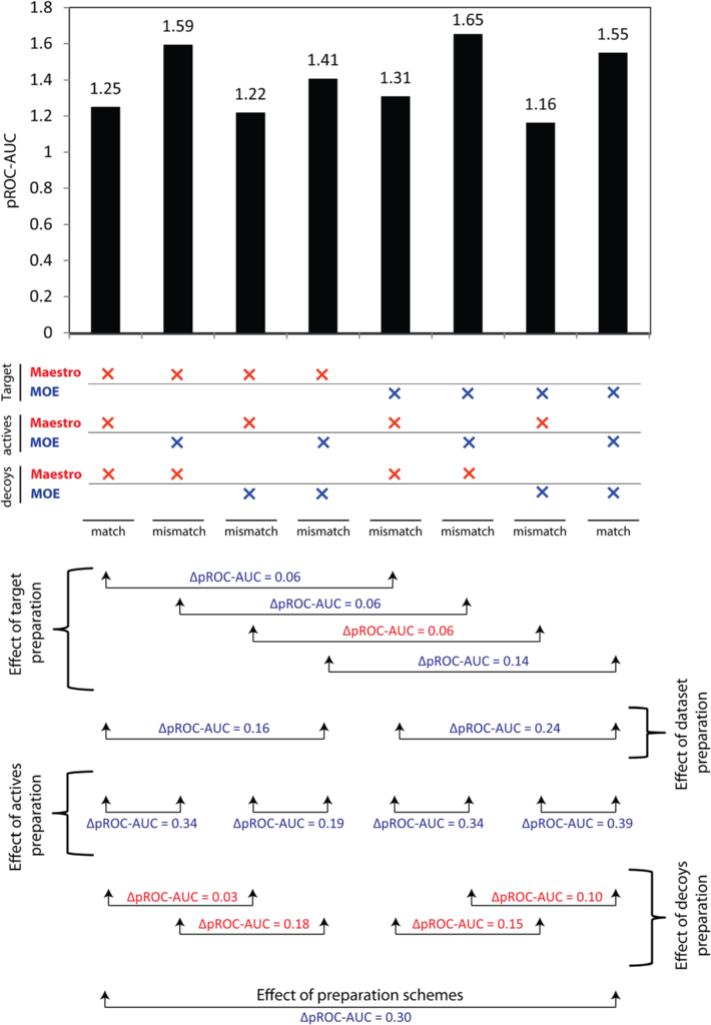
Match vs. mismatch docking assessment. Match vs. mismatch docking assessment of ACE benchmark set. Red and blue crosses represent Maestro and MOE preparations, respectively

We compared “matched” vs. “mismatched” docking experiments to evaluate the contribution of the previously discussed factors to screening performance differences. Docking the dataset (bioactives and decoys) into the protein target prepared with the same preparation scheme is a “matched” experiment, whereas, different preparation schemes for target and dataset will be called a “mismatched” experiment. The results obtained by this docking procedure are summarized in Fig. 1 and will be discussed subsequently.

### Impact of bioactives preparation on the docking rank and score

From Fig. 1, it is clear that preparation of the bioactives of ACE had the highest impact on the screening performance, while different preparation of decoys and target structure was less crucial. In contrast to the decoy set, the bioactive set preparation with MOE was always beneficial. The target structure preparation caused only small performance differences, typically in favor of MOE.

**Fig. 2:**
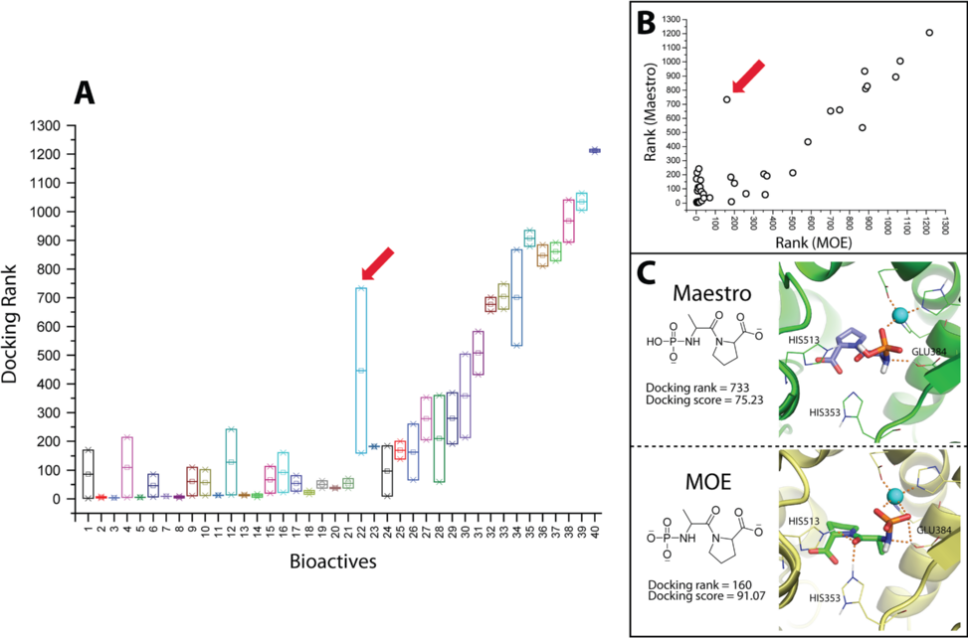
Docking rank comparison between Maestro and MOE preparations (for ACE bioactives). **a** Box plot illustrating the docking rank of Maestro and MOE prepared bioactives. The red arrow points towards the bioactive that shows the maximum difference in docking rank. **b** Scatter plot of the score-ordered rank of the respective bioactives prepared by Maestro vs. MOE preparation. **c** 2D representation and the docking pose of the bioactive showing maximum difference in docking rank between the two preparations. The docking rank and score are illustrated in the figure for this bioactive

Since the bioactives of ACE showed the highest influence on the screening performance, we tried to determine the possible factors responsible for this effect. We noticed that 35 % of the bioactives (14 out 40) showed better score-ordered rank for MOE vs. 65 % for Maestro preparation. Although there are less bioactives that exhibited preference towards MOE preparation, most of them are found predominantly at very low rank numbers, differing substantially from the Maestro rank numbers (Fig. 2b). This observation is sufficient to explain the superior performance of the MOE preparation for ACE, when using GOLD. To better visualize such perturbations in the score-ordered rank of the bioactives between the two preparations, a box plot of ranks of the respective bioactives influenced by the two preparations is shown in Fig. 2a. Interestingly, the maximum difference in the rank (red arrow) is attributable to a bioactive exhibiting different protonation states between the two preparations. The MOE-based protomer reproduced the key polar interactions with additional favorable H-bonding contacts in the binding site of ACE (as shown in Fig. 2c). Therefore, both the rank and the absolute score (ChemPLP) are superior for this protomer.

**Fig. 3:**
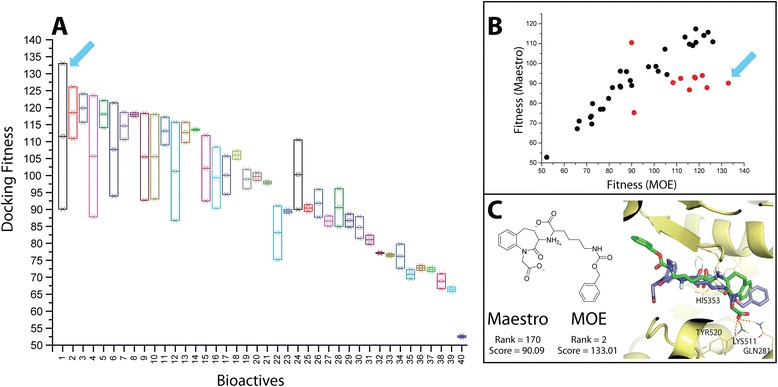
Docking score comparison between Maestro and MOE preparations (for ACE bioactives). **a** Box plot illustrating the docking fitness (score) of Maestro and MOE prepared bioactives. The blue arrow points towards the bioactive that shows the maximum difference in docking fitness. **b** Scatter plot of the docking fitness of the respective bioactives prepared by Maestro vs. MOE preparation. The red dots represent 10 compounds for which the docking fitness deviates more strongly between the two preparation schemes. **c** 2D representation and the docking pose of the bioactive showing maximum difference in the docking fitness between the two preparations. The best pose of the Maestro-prepared ligand is colored purple, the best posed of the MOE-prepared ligand is colored green. The docking rank and score are presented for this bioactive as well

In general, we observed that ~ 57 % of the bioactives (23 out 40) showed docking score (fitness) preference towards MOE preparation. To better investigating the effect of the two preparations on the docking score, we compared the docking score of the respective bioactives between the two preparations as shown in Fig. 3b. A trend of a mutual increase in fitness between the two preparations is visible; however, a subset of bioactives clearly favored the MOE preparation. For these high fitness value are reached when preparing them by MOE, while only mediocre fitness values are obtained for the respective Maestro-prepared bioactives. This small group of compounds causes the docking performance to improve always, when switching the bioactives preparation from Maestro to MOE in the match vs. mismatch assessment of Fig. 1. The box plot (Fig. 3a) shows that the bioactive molecule with the highest docking fitness when prepared with MOE (blue arrow), also showed the maximum difference in the docking score (Δ fitness) between the two preparations. The difference in the fitness value in this case is not attributable to a difference in protonation state. It is worthy to mention that this bioactive is quite flexible (number of rotatable bonds = 13). This flexibility is a challenging task for the conformational sampling during a docking run to find a reliable solution [39]. The docking pose of MOE appears to be a more reliable solution, because it reproduced the correct interaction pattern in the binding site of ACE, while the pose obtained by the Maestro-prepared ligand showed a 180° flip and, therefore, lost the key interaction pattern (as seen in Fig. 3c).

### Impact of dataset (bioactives vs. decoys) protonation

**Fig. 4:**
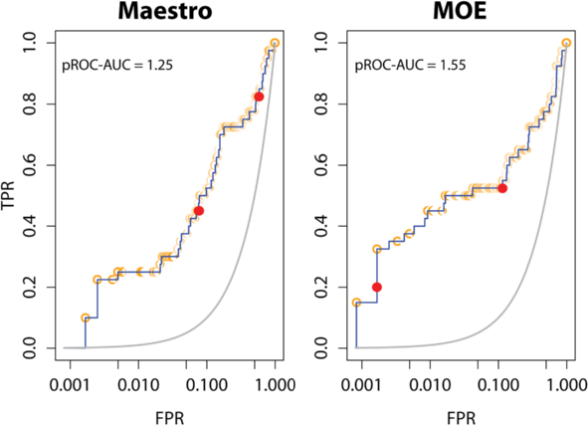
pROC plots of docking performance of the Maestro and MOE prepared ACE benchmark sets. Red (solid) and orange circles represent bioactives and decoys protomers, respectively. The 2D structural representation of the protomers of the respective bioactives is shown in Additional file 1: Figure S4 in SI

Only two of forty ACE bioactives (5 %) showed different protonation states between MOE and Maestro, while the decoy set showed 435 differences out of 1200 (36 %) between the two preparations. The mean value and distribution profile of the score-ordered rank, as well as the docking score, of the different protomers in the decoys does not deviate significantly between MOE and Maestro preparations, as seen from Table 2. These observations can also be directly seen from the respective pROC plots in Fig. 4: no changes in the occurrence of the different decoy protomers are visible in the early recognition regions of the pROC plots. On the other hand, the two protomers of the bioactives clearly show higher differences in either the score-ordered rank or the docking score between the two preparations. Thus, it is unlikely that the decoys protonation difference between the two preparations would exert higher impact than the bioactives protonation.

**Table 2:** Basic statistics showing the distribution of Maestro and MOE protomers for the respective docking runs

	MOE				Maestro			
Bioactives (n = 2)	Mean	SD	Min	Max	Mean	SD	Min	Max
Rank	85.5	NA	11	160	421.5	NA	110	733
Docking score	104.70	NA	91.07	118.30	83.97	NA	75.23	92.72
Decoys (n = 435)								
Rank	648.6	356.5	8	1240	646.7	358.3	7	1240
Docking score	77.33	12.34	39.19	120.80	75.63	12.28	39.24	112.30

**Fig. 5:**
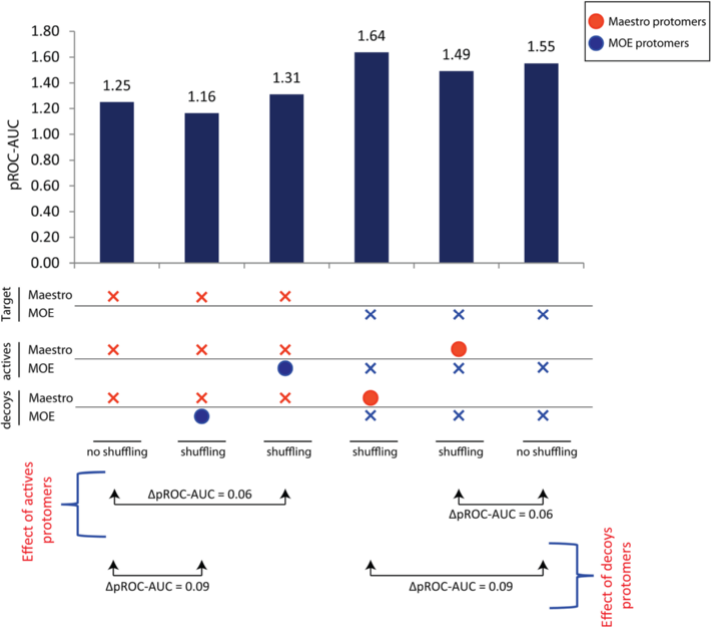
Shuffling vs. no shuffling assessment. Shuffling vs. no shuffling assessment for Maestro and MOE protomers showing the impact of the protomers of the bioactives vs. decoys

To quantify the impact of the protonation of the dataset, we conducted a shuffling experiment. The idea of this experiment is to replace only the protomers selected by one preparation scheme (e.g. MOE) with the generated protomers from the other preparation scheme (e.g. Maestro) while keeping the rest of the input data from the original preparation (e.g. MOE). This assessment will result in a new score-ordered list with new values of the replaced protomers and subsequently a new pROC-AUC. By calculating the ΔpROC-AUC values after shuffling of the protomers, one is able to evaluate to which extent the protomers have an impact. Comparing the shuffling impact of both bioactives and decoys protomers, we found that the pROC-AUC values only change marginally by 0.06 and 0.09 for bioactives and decoys protomers, respectively (Fig. 5). This highlights that the different protomers do not give a sufficient explanation for the initially observed pROC-AUC difference of 0.3.

Upon totally deleting the two bioactives with different protomers and recalculating the pROC-AUC, we observed only non-significant differences (0.03 for Maestro and and 0.02 for MOE preparations). Still the difference between MOE and Maestro preparations in this case is significant (ΔpROC-AUC = 0.26). This observation suggests that the protonation state selection between the two preparations is only a minor factor and other factors (e.g. geometric features) should contribute much more to the observed performance differences.

### Impact of bioactives geometric features?

**Fig. 6:**
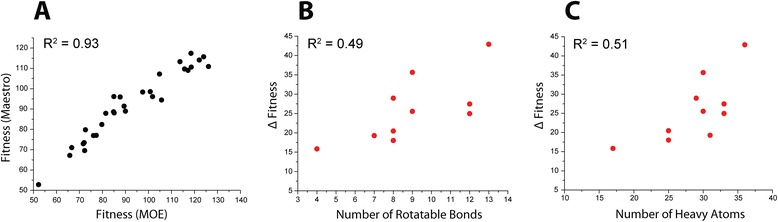
Docking score behavior of ACE bioactives. **a** Scatter plot of the docking fitness of the 30 bioactives prepared by Maestro vs. MOE after isolating a subset of 10 outliers. **b** Scatter plot of the docking fitness of the subset of isolated 10 outliers plotted against their number of rotatable bonds and **c** plotted against their respective number of non-hydrogen (heavy) atoms

As observed from Fig. 3, a highly flexible bioactive ligand showed a maximum fitness difference between the two preparations. Therefore, to investigate whether the flexibility of the bioactives is a relevant factor contributing to the screening performance difference between the two preparations, we compared the docking score difference between MOE and Maestro preparations (Δfitness) to the number of rotatable bonds of the respective bioactives. No correlation could be observed when all bioactives were included in this comparison (data in Additional file 1: Figure S5 in SI). Despite the absence of a general trend, we found that upon isolation of a subset of outliers (10 bioactives with red dots in Fig. 3b) from the correlation of the docking scores of MOE vs. Maestro preparations, this isolated subset showed some tendency (Fig. 6) to increase Δfitness with the number of rotatable bonds (R^2^ = 0.49) and the number of non-hydrogen (heavy) atoms (R^2^ = 0.51). The remaining set of 30 bioactives shows a striking correlation (R^2^ = 0.93) between the docking scores of the MOE and Maestro preparations. Hence, the docking performance of 75 % of bioactives is not much influenced by the preparation scheme, while in the subset (25 %), higher flexibility and size of the compound can explain the observed differences partially. Still, it should be noted that the mediocre R^2^ of about 0.5, implies that flexibility and size not necessarily increase Δfitness, but that other important factors can affect the docking score and performance, e.g. flexible ring conformation and input conformation geometry.

**Fig. 7:**
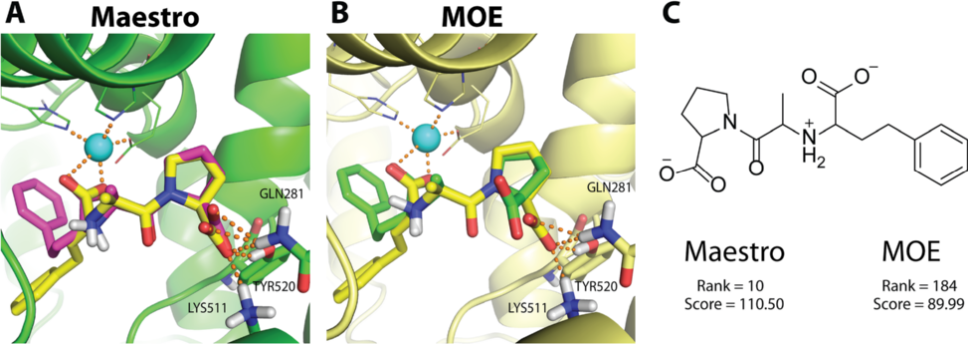
Impact of flexible ring conformation (ACE bioactives). Conformation of enalaprilat – (crystal structure in yellow sticks) in complex with ACE (PDB code: 1UZE) compared to docking results achieved after ligand preparation with Maestro (**a**) and MOE (**b**). Purple and green sticks indicate the docked poses of enalaprilat by GOLD. **c** 2D representation of enalaprilat with docking score and rank difference indicated below

It is worthy to mention that the majority of the bioactives of ACE (35 out of 40) possess five to seven-membered flexible ring systems. We observed that all of the respective bioactives of the previously mentioned subset of 10 bioactives displayed different selection of their ring conformations between the two preparations while only 7 out of the remaining 30 bioactives showed different selection. This also highlights that the high Δ fitness values between the two preparations can also be attributed to the selection of different ring conformations. Searching the PDB for crystal structure of ligands in complex with ACE, we found one example (enalaprilat) [40] that is part of our subset of 10 bioactives (Fig. 7). Despite the fact that the co-crystallized pyrrolidine conformation of enalaprilat is almost planar and hard to be predicted, the pyrrolidine conformation produced by Maestro preparation was – to a certain extent – comparable to it and conserved the key polar contacts of enalaprilat’s carboxylate group with the surrounding polar residues after docking. However, in the MOE preparation, the selected pyrrolidine conformation is different and led to an obvious deviation in the placement of enalaprilat’s carboxylate group, thus, making close contacts with the surrounding polar residues infeasible. Consequently, the docking score and rank was superior for Maestro in this case. Although this is just one example, it highlights that the ring conformation selection by the preparation procedures has an important, sometimes crucial, effect on reproducing the correct poses and, hence, on the screening performance.

To monitor differences in the input conformations between the two preparations of the bioactives, we calculated the pairwise RMSD for each bioactive molecule. We found a trend for bigger bioactives to show higher RMSD values between their input conformations (R^2^ = 0.52, see Additional file 1: Figure S6 in SI). This certainly makes sense, since it is challenging for the two force fields to find closely related energy minima, when molecular size and flexibility increases. However, we did not observe any reasonable trend (R^2^ always below 0.2) between the pairwise RMSD of the bioactive molecules and the Δfitness in any of the 18 datasets. It is evident from other studies in the literature [31, 32] that small changes in the input conformation of the molecule (e.g. torsional angles and bond lengths) can affect the docking performance to a vast extent. Thus, we cannot rule out that differences in the way the input conformations were generated in the two procedures will cause a significant influence on the different docking performances observed.

### Impact of decoys preparation

**Fig. 8:**
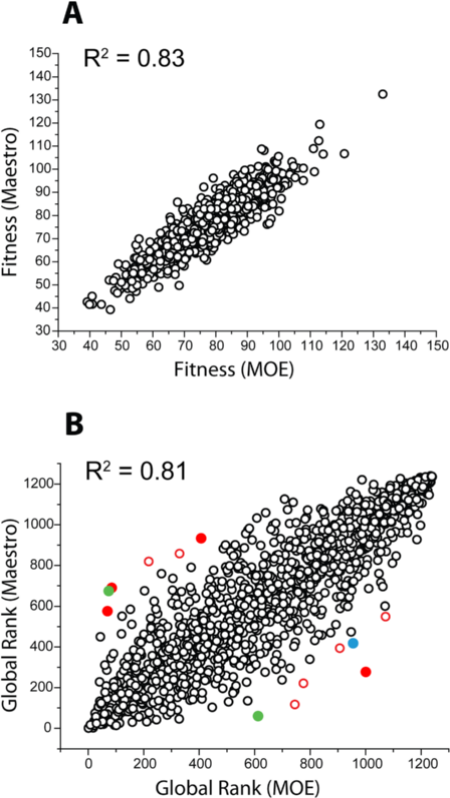
Docking score and rank behavior of the ACE decoys. **a** Scatter plot of the docking fitness of the respective 1200 decoys prepared by Maestro vs. MOE preparation. **b** Scatter plot of the global docking rank of the respective 1200 decoys prepared by Maestro vs. MOE preparation. The colored dots and circles are the decoys that showed Δdocking rank > 500. Colored dots represent decoys that show different protonation (red), tautomerization (blue) or ring conformations (green) between the Maestro and MOE preparations, whereas the red circles represent decoys that show none of the previous differences

Although the decoys showed less impact on the screening performance compared to the bioactives, we have scrutinized the differences between the two preparations in more detail. Figure 8a highlights that there is a mutual increase in the docking score (fitness) of the respective decoy between MOE and Maestro preparations (R^2^ = 0.83). This suggests that the Δfitness values between the two preparations are quite small. Generally, we observed that 60 % of the decoys (720 out 1200) showed docking score preference toward the MOE preparation scheme. This can explain the fact that the docking performance (i.e. pROC-AUC) always improved when switching the decoys preparation from MOE to Maestro in the match vs. mismatch assessment in Fig. 1, since higher docking scores for the majority of the decoys with MOE preparation would worsen the separation between the bioactives and decoys, and as a consequence lower pROC-AUC values for experiments with MOE prepared decoys.

Transforming the docking score information into the global docking rank (i.e. including the bioactives’ ranks) showed more scattering of some decoys around the fitted line (R^2^ = 0.81) for the rank correlation, as seen in Fig. 8b. For getting some answers about the possible factors that affected the difference in the docking rank between the two preparations for the decoys, we first looked at the most obvious outliers. Thus, we applied a cutoff value of the docking rank difference of 500 (i.e. Δdocking rank > 500) bearing in mind that the maximum Δdocking rank difference observed for the bioactives was 573. We ended up with 13 decoys that showed a Δdocking rank between the two preparations above the cutoff. In summary these 13 decoys exhibit the following properties: (a) four decoys showed differences in the protonation state, (b) one decoy had an altered tautomer state, (c) two decoys deviated in their ring conformations, and (d) six decoys did not show any of previous factors, but three of those had a rather high number of rotatable bonds (more than 8). It is noteworthy that the maximum Δdocking rank of the decoys is attributable to a difference in protonation state between the two preparations. Generally, we noticed that 49 % of the decoys (586 decoys) showed better ranks after the MOE preparation, while 51 % had better ranks after the Maestro preparation. Because this ratio is almost exactly 1:1, it does not provide any clues for understanding why the docking performance for ACE is superior in case of the MOE preparation. This indicates that the docking rank of the bioactives is certainly much more important for the overall result than the docking rank of the decoys. We conclude that similar aspects as discussed before for the bioactives cause differences between the two preparations, but the overall influence of such differences in the decoys is significantly inferior.

### Impact of target structure preparation

**Fig. 9:**
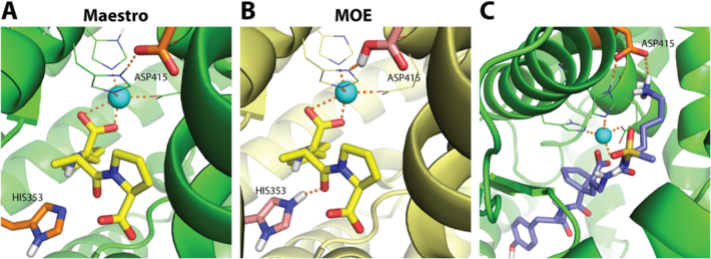
Impact of the protein target preparation. Binding site of ACE (PDB code: 1UZE) prepared by Maestro (**a**) and MOE (**b**). The co-crystallized ligand is represented as yellow sticks. Asp415 and His353 show different protonation states in (**a**) and (**b**). **c** A relatively large bioactive compound (purple sticks) forms hydrogen bonds with Asp415 in the binding site prepared with Maestro

Interestingly, the preparation schemes yielded different protonation states of binding site residues His353 and Asp415 as shown in Fig. 9. Unlike Maestro preparation, MOE protonated the residues His353 and Asp415 in the binding site of ACE. The charged nitrogen of HIS353 pointed towards the co-crystallized ligand. Although, Asp415 is located not in close proximity of the zinc ion and the co-crystallized ligand, it could be accessible by larger molecules, as shown in Fig. 9c. These differences between MOE and Maestro protonation for these residues contribute to a difference in hydrogen bonding capabilities of the binding site. Another important factor for target-dependent screening performance differences is the complexity of the microenvironment of metal-containing targets [29, 38]. Interactions of ligands with metal ions can be recognized by the docking program only for certain ligand protomers or tautomers [38]. This phenomenon is not trivial to handle since selecting a reasonable protomer/tautomer in an automated fashion is not trivial.

### Score normalization

**Fig. 10:**
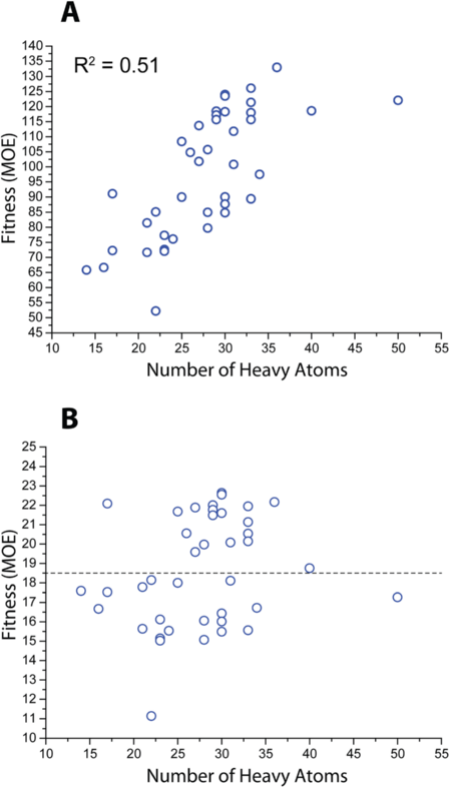
Score normalization behavior of ACE bioactives. **a** Scatter plot of the docking fitness of the respective bioactives prepared by MOE vs. number of non-hydrogen (heavy) atoms, and **b** after normalizing the docking fitness by N^1/2^. The dashed line represents the mean value of the normalized fitness

The employed docking tools (GOLD and Glide) in this study utilize empirical scoring functions. Empirical scoring functions are additive and generally demonstrate a dependence on molecular weight. This results in a bias toward heavier molecules being ranked at the top of a score-ranked list of molecules [41, 42], as can also be seen for the bioactives (e.g. MOE preparation) of the ACE example, in Fig. 10a. Weighing the docking score is a well-known strategy to reduce the molecular weight bias in VS [39]. For this we divided the docking score by the square root of the number of heavy atoms (N^1/2^) or the cubic root of the squared number of heavy atoms (N^2/3^) [41]. For ACE, normalizing fitness values of the bioactives (e.g. MOE preparation) produced a random distribution around their mean value. This eliminated the biased distribution of their original values as well as enhanced the screening performance, as shown in Table 3.

**Table 3:** Overview of the screening performance based on the normalized scores obtained by GOLD docking after MOE preparation. Further data on the normalization of the docking performances for Glide and GOLD after MOE and Maestro preparation are available in Additional file 1: Table S4 in the Supporting Information

Target	N^2/3^	ΔpROC-AUC_*N*_^*a*^	N^1/2^	ΔpROC-AUC_*N*_	MW^*c*^ mean per dataset	NHA^*d*^ mean per dataset
ACE	1.76	*0.21* ^*b*^	1.81	*0.26*	398.7	27.6
ACHE	0.64	*−0.07*	0.71	−0.01	398.6	28.6
ADRB2	0.76	0.02	0.80	*0.07*	425.5	29.7
CATL	0.55	*−0.23*	0.63	*−0.16*	482.0	33.9
DHFR	1.07	*0.32*	1.02	*0.27*	385.8	26.8
ERBB2	1.00	*−0.85*	1.34	*−0.51*	473.8	33.8
HDAC2	0.93	*−0.09*	1.03	0.00	388.4	27.5
HIVPR	0.75	*−0.70*	0.97	*−0.48*	584.3	41.2
HSP90	0.58	*0.16*	0.55	*0.12*	424.1	29.3
JAK3	0.76	−0.02	0.82	0.04	404.1	29.0
JNK2	0.68	*−0.09*	0.71	−0.05	413.5	29.7
MDM2	0.35	*−0.16*	0.40	*−0.11*	554.7	36.4
P38	0.53	0.04	0.53	0.04	383.1	27.7
PI3KG	1.02	0.02	1.17	*0.16*	412.3	29.4
PNP	1.22	*0.24*	1.24	*0.26*	269.1	18.9
PPARg	0.83	−0.02	0.89	0.04	463.6	32.6
Thrombin	1.12	*−0.11*	1.20	−0.03	479.7	33.5
TS	1.06	*−0.21*	1.26	−0.01	415.3	29.5

^*a*^ΔpROC-AUC_*N*_ is calculated as (pROC-AUC of normalized – pROC-AUC of original). pROC-AUC of original values are present in Table 1. ^*b*^Italicized values demonstrate significant ΔpROC-AUC_*N*_ (i.e. -0.05 > ΔpROC-AUC_*N*_ > +0.05) where negative and positive values highlight inferior and improved performance by normalization, respectively. Regular-formatted values demonstrate non-significant change by normalization. ^*c*^MW is the molecular weight. ^*d*^NHA is the number of heavy atoms

In general, we found that the GOLD screening performance for many datasets (see Table 3) was improved when applying the docking score normalization. In contrast, the Glide docking performance was reduced for almost all benchmark sets when applying the score normalizations (data in Additional file 1: Table S4 in SI). We found that the N^2/3^ normalization, which emphasizes the number of heavy atoms more strongly than N^1/2^ in the final score, yielded worse performance results than the N^1/2^ normalization. Interestingly, GOLD docking into HSP90 with the MOE preparation displayed a pROC-AUC < 0.434 – worse than random performance – with the original setup, but gave a significantly improved enrichment for both N^2/3^ and N^1/2^ normalizations and the screening performance was shifted to be better than random (>0.434). It is obvious that for several targets with a larger average size (e.g. average number of heavy atoms or molecular weight) of their datasets (e.g. HIV1PR and ERBB2), their performance after normalization was penalized quite significantly, and vice versa for targets that show a relatively small average size (e.g. PNP), their performance after normalizations has been improved (Table 3).

Based on our results, we would recommend that the medicinal/computational chemists may test the performance of the N^1/2^ normalization for GOLD (ChemPLP), whereas for Glide, no normalization appears to be required. This may not be surprising, since the ChemPLP scoring function of GOLD has been originally developed for pose prediction purposes [43, 44], while Glide SP had been optimized for screening enrichment and docking accuracy [45, 46].

## Methods

### Preparation of targets

Maestro preparation scheme: For each crystal structure the coordinates were retrieved from the Protein Data Bank (PDB) in our dataset. A comprehensive list of PDB codes is given in Additional file 1: Table S1. The preparation of the original PDB files was conducted by assigning bond orders, adding explicit hydrogens, creating zero-order bonds to metals, and converting selenomethionines to methionines using the Protein Preparation Wizard in Maestro (version 9.1) [47]. Redundant and identical macromolecule chains with non-essential co-factors, ions, water molecules and ligands were discarded. Exceptions were made for (PDB code: 1i00) with a co-substrate in the ligand binding site, and for structures that contained metal ions (e.g. Zn^2+^, and Mg^2+^) in their ligand binding pocket (PDB code: 1uze and 3max). The metal binding states were generated at pH 7.0 by Epik [48, 49]. H-bond network optimization, protonation and tautomeric states of Asp, Glu, Arg, Lys and His were sampled by “ProtAssign” in the standard mode. Possible alternative orientations of Asn and Gln residues were also generated. The native geometry of the binding sites was preserved without in-place ligand-protein minimization. Prepared structures were saved as MAE and PDB files. The MAE files were used for Glide [45, 46] (version 5.6) docking, the PDB files were used for GOLD (version 5.1) docking [50–53].

MOE preparation scheme: Again, identical and redundant protein chains with non-essential co-factors, ions, water molecules and ligands were discarded while co-substrates, and metal ions in the ligand binding site were preserved. Bond orders, formal charges and explicit hydrogens were added to the complex structure. Subsequently, protonation and tautomeric states were generated, and optimization of the H-bond network of the protein-ligand complexes was performed with the MOE ‘Protonate 3D’ function at standard settings (T = 300 K, pH = 7.0, ionic strength I = 0.1 mol/l). Prepared structures were saved as pdb files and then used for Glide and GOLD. Again, the native geometry of the binding sites was preserved without in-place ligand-protein minimization. An overview of the procedures employed by Maestro and MOE schemes is shown in Table 4.

**Table 4:** Overview of Maestro and MOE preparation schemes for targets and datasets. This table shows comparable settings for preparing the input

	Maestro	MOE
1) Targets preparation	ProtAssign	Protonate 3D
Adding hydrogens and H-bond optimization	“standard”	“default”
Asn and Gln flipping	“standard”	“default”
Protonation/tautomerization states of Asp, Glu, Arg, Lys and His	“standard”	“default”
Number of protomer/tautomer retrieved for Asp, Glu, Arg, Lys and His	one	one
2) Dataset (bioactives and decoys) preparation	Ligprep	wash and minimize
Minimization	yes	yes
Number of protomer/tautomer retrieved	one	one
Input chirality retained	yes	yes

### Preparation of DEKOIS 2.0 datasets

Maestro preparation scheme: All molecules of the datasets were prepared by LigPrep (version 2.4) [54]. The molecules were minimized using the OPLS-2005 force field. The protonation state was generated at pH 7.0 for each molecule. The specified stereoconfiguration of all bioactives and decoys of the datasets was retained. All prepared molecules were saved as SD files for Glide and GOLD docking. MOE preparation scheme: “molecule wash” function was used to generate meaningful protonation states by deprotonating strong acids and protonating strong bases. Energy minimization of all molecules was then performed using the MMFF94x force field at a gradient of 0.01 RMSD (i.e. if the gradient falls below RMSD, the minimization stops). Existing chirality was preserved and partial charges were calculated according to the standard parameters of the force field.

We conducted the pairwise RMSD calculation for the respective molecules of the datasets between Maestro and MOE schemes by employing in-house *python* script applying the “smart_rms” function of GOLD.

### Docking experiments

The docking runs were performed with Glide and GOLD at the default settings. Only the best pose per molecule was retrieved for the molecules of the datasets from the docking and pROC-AUC values were calculated thereof. For Glide (version 5.6) docking, we generated the receptor grid-box by default settings, with an approximate size (on average) 20 x 20 x 20 angstroms for most of the targets. We conducted the standard precision docking mode (SP) and also considered Epik state penalties for metal-containing binding sites. By default, the cutoff for keeping initial docking poses was 100.0 kcal/mol (relative to the best scored initial pose). The best five final docking poses per molecule were selected for post-docking minimization within Glide. For GOLD (version 5.1) docking, residues of the binding site were defined by specifying the crystal structure ligand coordinates and using a cutoff radius of 10 Å, with the ‘detect cavity’ option enabled. The scoring function used for GOLD docking experiments was ChemPLP. The search efficiency of the genetic algorithm was kept at the standard 100 % setting. The docking was early terminated when the top three solutions were within 1.5 Å RMSD.

In order to avoid over-interpreting small changes in the docking performance and to account for the heuristic nature of the docking tools, we defined the “safety margin” of ±0.05 ΔpROC-AUC_*N*_ below which it is considered non-significant in the normalization task. This ΔpROC-AUC_*N*_ is calculated as:

$$ \Delta \mathrm{pROC}-\mathrm{A}\mathrm{U}{\mathrm{C}}_N=\mathrm{pROC}-\mathrm{A}\mathrm{U}\mathrm{C}\left(\mathrm{normalized}\right)-\mathrm{pROC}-\mathrm{A}\mathrm{U}\mathrm{C}\left(\mathrm{original}\right) $$

## Conclusion and outlook

We have shown in this study that application of comparable setups for the preparation of targets, bioactives and decoys by different modeling packages (Maestro or MOE) affected the screening performance of Glide and GOLD in the majority of the investigated targets. These differences in the screening performance are attributable to multiple factors, such as the different selection of: (a) the protonation/tautomerization state of the target’s binding site residues and molecules of the datasets, and (b) the input geometry of the prepared molecules due to the intrinsic differences between the force field parameters of both preparation schemes. Match versus mismatch docking runs of the ACE dataset as a case example demonstrated that the preparation of the bioactive molecules has the highest impact on the screening performance. The impact of the protonation state differences in the decoys or bioactives was assessed by a shuffling experiment. In both cases the importance of the protonation state differences seemed small, yet slightly more relevant for the bioactive molecules. Having a look at the docking rank and score of the respective bioactive between the two preparations, we found that the maximum difference of the rank is attributable to a bioactive ligand that showed a difference in its protonation state between the two preparations. The maximum fitness difference (Δfitness) was caused by a bioactive ligand that showed a highly flexible structure. Analyzing the geometric features as possible impact factors on the screening performance difference, we found that the flexibility features (e.g. number of rotatable bonds and number of heavy atoms) showed a reasonable correlation (R^2^ = 0.49 and 0.51, respectively), with the Δfitness for a subset of ten bioactives, which exhibited the highest deviations in the fitness values. For the remaining 30 bioactives, fitness values between the two preparations were highly similar (R^2^ = 0.93). Among the geometric features identified is also the impact of selecting different conformations of flexible rings in bioactives as well as the input conformation of the dataset for docking. Analysis of the decoys behavior between the two preparation schemes agreed widely with the general findings observed for the bioactives.

**Fig. 11:**
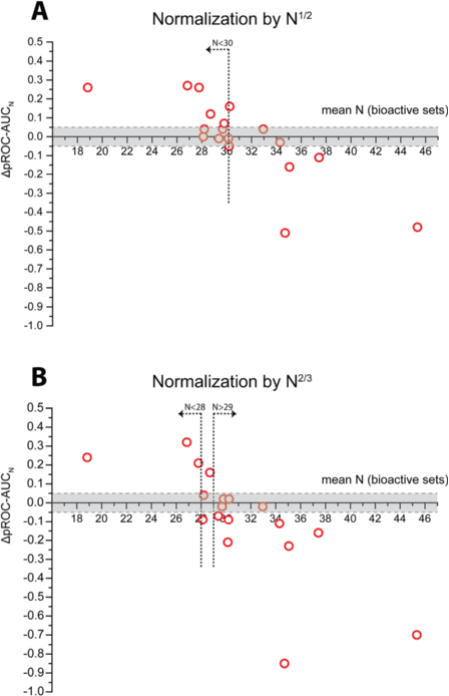
Behavior of the score normalization of GOLD docking (MOE preparation) with the mean number of heavy atoms per bioactive set. **a** Scatter plot of ΔpROC-AUC_*N*_ vs. the mean number of heavy atoms (N) per bioactive set for N^1/2^, and **b** for N^2/3^. The grey region of ΔpROC-AUC_*N*_ indicates the non-significant margins (±0.05)

We recommend that computational/medicinal chemists should evaluate the available docking programs with several different preparation/docking setups – e.g. using DEKOIS 2.0 – to retrieve the best possible performance which then can be used for an actual SBVS effort. In addition, score normalization strategies eliminated the biased fitness shown by GOLD for the larger bioactives of ACE. Generalizing these normalization strategies on the 18 targets, we observed improved performances for the majority of the GOLD docking results, whereas normalization led to detrimental performances for the majority of Glide docking runs. Therefore, we recommend that computational/medicinal chemists should employ score normalization strategies for GOLD (ChemPLP) especially using a normalization factor of N^1/2^ for bioactive sets with a mean number of heavy atoms of 30 or below (see Fig. 11). In particularly these cases, we have observed an increased chance that pROC-AUC values are improved. In none of the studied datasets of this group, normalization by a factor of N^1/2^ produced significantly worse results. However, for bioactive sets with a larger mean number of heavy atoms, the risk of detrimental effects on the pROC-AUC values is obvious. For using a normalization factor of N^2/3^, we observed a higher chance for improvements below a mean number of heavy atoms of 28 and a high risk for inferior performance above a mean number of heavy atoms of 29. We propose that these numbers could be used as rough guidelines and should be validated by using more benchmark sets in the future. In general, testing normalization strategies for empirical scoring functions that have been mainly optimized for pose prediction purposes will be useful.

Elucidating the effect of two different preparations on the screening performance and deconstructing the possible factors behind these performance differences is certainly useful for making reasonable decisions in virtual screening campaigns. In addition, another important factor for understanding benchmarking results can be the composition of the set of bioactives with respect to the frequent occurrence of similar scaffolds or common substructures. For achieving this, we aim at studying the impact of the two preparation schemes on the chemotype recognition during the docking process in a subsequent publication.

## Additional file

Additional file 1:
**(Supporting Information): Detailed insights with additional Tables and Figures.** We provide additional Tables and Figures to give more detailed insights into: Overview of the targets description (Table S1), Overview of the difference in protonation/tautomerization states of the binding site residues of the respective targets prepared by the two preparations (Table S2), Overview of protonation/tautomerization state difference between the two preparations as well as the average number of the rotatable bonds, heavy atoms and pairwise RMSD per target (Table S3), Overview of the normalization strategies on GOLD and Glide dockings on MOE and Maestro preparation schemes (Table S4), Scatter plot of the correlation between the percentage difference in protonation/tautomerization states of the bioactives and the absolute value of the ΔpROC-AUC (Figure S1), Binding site of DHFR showing the difference between the two preparations and the match vs. mismatch assessment for DHFR benchmark sets using Glide (Figure S2), Overview of the protonation/tautomerization states selected by the two preparations for DHFR bioactive set (Figure S3), Overview of the protonation/tautomerization states selected by the two preparations for ACE bioactive set (Figure S4), Scatter plot of the correlation between Δ fitness vs. number of rotatable bonds (Figure S5), and scatter plot of the correlation between the number of heavy atoms and the pairwise RMSD of the two conformations resulted from the two preparations for ACE bioactives (Figure S6).
