# Supplementary material for: Applying DEKOIS 2.0 in structure-based virtual screening to probe the impact of preparation procedures and score normalization
Source: J Cheminform. 2015 May 20;7:21. doi: 10.1186/s13321-015-0074-6 (PMC4450982; doi:10.1186/s13321-015-0074-6)
Supplement: Additional file 1: — (Supporting Information): Detailed insights with additional Tables and Figures. We provide additional Tables and Figures to give more detailed insights into: Overview of the targets description (Table S1), Overview of the difference in protonation/tautomerization states of the binding site residues of the respective targets prepared by the two preparations (Table S2), Overview of protonation/tautomerization state difference between the two preparations as well as the average number of the rotatable bonds, heavy atoms and pairwise RMSD per target (Table S3), Overview of the normalization strategies on GOLD and Glide dockings on MOE and Maestro preparation schemes (Table S4), Scatter plot of the correlation between the percentage difference in protonation/tautomerization states of the bioactives and the absolute value of the ΔpROC-AUC (Figure S1), Binding site of DHFR showing the difference between the two preparations and the match vs. mismatch assessment for DHFR benchmark sets using Glide (Figure S2), Overview of the protonation/tautomerization states selected by the two preparations for DHFR bioactive set (Figure S3), Overview of the protonation/tautomerization states selected by the two preparations for ACE bioactive set (Figure S4), Scatter plot of the correlation between Δ fitness vs. number of rotatable bonds (Figure S5), and scatter plot of the correlation between the number of heavy atoms and the pairwise RMSD of the two conformations resulted from the two preparations for ACE bioactives (Figure S6). [file 13321_2015_74_MOESM1_ESM.pdf]

Additional file 1 (Supporting Information): Detailed insights with additional Tables and Figures

Applying DEKOIS 2.0 in Structure-Based Virtual Screening to Probe the Impact of Preparation Procedures and Score Normalization

Tamer M. Ibrahim[a,b], Matthias R. Bauer[a] and Frank M. Boeckler[a§]

[a]Laboratory for Molecular Design and Pharmaceutical Biophysics, Department of Pharmaceutical and Medicinal Chemistry, Institute of Pharmacy, Eberhard Karls University Tuebingen, Auf der Morgenstelle 8, 72076 Tuebingen, Germany

[b]Department of Pharmaceutical Chemistry, Faculty of Pharmacy and Biotechnology, German University in Cairo, Cairo 11835, Egypt

[§]Corresponding author

Email addresses:

TMI: tamer.abdelrehim@uni-tuebingen.de

MRB: mbauer@mrc-lmb.cam.ac.uk

FMB: frank.boeckler@uni-tuebingen.de

Table of Contents:

Table S1. Overview of the targets description. 3

Table S2. Overview of the difference in protonation/tautomerization states of the binding site residues of the respective targets prepared by the MOE and Maestro preparation schemes. 4

Table S3. Overview of protoantaion/tautomerization states difference between MOE and Maestro schemes as well as the average number of the rotatable bonds, heavy atoms and pairwise RMSD per target. 8

Figure S1. Scatter plot of the correlation between the percentage difference in protonation/tautomerization states of the bioactives and the absolute value of the ΔpROC-AUC. 9

Figure S2. Binding site of DHFR showing the difference between Maestro (A) and MOE (B). (C) Match vs mismatch assessment for DHFR benchmark sets using Glide. 11

Figure S3. Overview of the protonation/tautomerization states selected by MOE and Maestro preparations for DHFR active set 12

Figure S4. Overview of the protonation states selected by MOE and Maestro preparations for ACE bioactive set. 13

Figure S5. Scatter plot of the correlation between (Δ fitness) vs. (number of rotatable bonds). (A) When including the whole bioactives of ACE benchmark set. (B) When including cutoff > 7 number of rotatable bonds. 14

Figure S6. Scatter plot of the correlation between the number of heavy atoms and the pairwise RMSD of the two conformations resulted from Maestro and MOE preparations for ACE bioactives 15

Table S4. Overview of the normalized performance of GOLD and Glide runs with the two preparations. Green color is for (ΔpROC AUCN > +0.05), and purple color is for (ΔpROC AUCN < -0.05). The original pROC-AUC values are in Table 1 in the manuscript........ 16

Table S1. Overview of the targets description.

Target Name	PDB code	Description	Functional Class
ACE	1uze	Angiotensin-I-converting enzyme	Protease
ACHE	1eve	Acetylcholinesterase	Hydrolase
ADRB2	3ny9	Beta-2 adrenergic receptor	GPCR
CATL	3bc3	Cathepsin L	Protease
DHFR	1s3v	Dihydrofolate reductase	Oxido-Reductase
ERBB2	3pp0	Human Epidermal growth factor Receptor 2	Kinase
HDAC2	3max	Histone Deacetylase 2	Protease
HIV1PR	3nu3	HIV-1 protease	Protease
HSP90	1uy6	heat shock protein 90	Protease
JAK3	3lxl	Janus Kinase 3	Kinase
JNK2	3npc	Mitogen-activated protein kinase 9	Kinase
MDM2	3lbk	p53-Binding Protein MDM2	Ligase
P38-alpha	1ouk	P38 mitogen-activated protein kinases	Kinase
PI3Kg	3dbs	Phosphoinositid-3-Kinase gamma	Kinase
PNP	1b8o	Purine nucleoside phosphorylase	Transferase
PPARg	1fm9	Peroxisome proliferator-activated receptor gamma	Nuclear Receptor
THROMBIN	3rm2	thrombin	Protease
TS	1i00	Thymidylate Synthase	Transferase

Table S2. Overview of the difference in protonation/tautomerization states of the binding site residues of the respective targets prepared by the MOE and Maestro preparation schemes.

	MOE (prot3D)	Maestro (PPW-protassign)
ACHE	SER200 (OG)* proton has different orientation	ASN324 flipped (far from ligand)
	HIS440 NE is protonated and neutral	GLN74 flip (in BS, no interaction)
		HIS440 ND is protonated and neutral
ACE	ASP415 (OD2) is protonated and fipped near ligand (no direct interaction)	ASP415 (OD2) is deprotonated (no direct interaction)
	GLU384 (OE2) is protonated and forms HB with ligand	GLU384 (OE2) is protonated and forms HB with ligand
	HIS383 (NE2) is deprotonated and chelating with Zn	HIS383 (NE2) is deprotonated and chelating with Zn
	HIS387 (NE2) is deprotonated and chelating with Zn	HIS387 (NE2) is deprotonated and chelating with Zn
	His353 (HIP), (NE2) is protonated and neutral, ND1 is protonated, charged and making HB with the ligand	His353 (NE2) is deprotonated
ADRB2	THR110(HG1) is differently oriented.	
	THR118 (HG1) is differently oriented.	HIS269 flipped
	THR195 (HG1) is differently oriented.	HIS93 flipped
	SER204 (HG) is differently oriented.	HIS178 flipped
	SER207 (HG) is differently oriented.	
	TYR308 (HH) is differently oriented.	

	TYR316 (HH) is differently oriented.<br><br>HIS269 flipped	
CATL	HIS140 flipped, and (ND1) is protonated and positively charged<br><br>HIS163 (ND1) is protonated and positively charged<br><br>ASP114 is protonated	HIS140 flipped, and ND1 is protonated and positively charged<br><br>HIS163 ND1 is deprotonated and neutral<br><br>ASP114 is protonated<br><br>ASN66 flipped
DHFR	GLU30 (OE2) is protonated, making HB with ligand<br><br>GLN35 (NE2) flipped near to ligand	GLU30 (OE2) is protonated, making HB with ligand<br><br>GLN35 (NE2) flipped near to ligand<br><br>ASN64 flipped
ERBB2	SER728 (HG) is differently oriented.<br><br>THR798 (HG1) is differently oriented.	HIS809 flipped, NE2 is protonated and neutral
HDAC2	TYR29 (HH) is in different orientation<br><br>SER118 (HG) is differently oriented.<br><br>HIS146 (NE2) is protonated & neutral; (ND1) is protonated and positively charged (HIP, HIE)<br><br>TYR308 (HH) is differently oriented.	HIP184 for both moe and protassign (ND1 is charged and protonated)<br><br>HIS146
HIV1PR	THR31 (HG1) is differently oriented, far from ligand<br><br>THR131 (HG1) is differently oriented., far from ligand	nothing special
HSP90	ASN51 (ND2) flipped	ASN51 (ND2) not flipped

	SER52 (HG) is differently oriented.<br><br>TYR139 (HH) is differently oriented.<br><br>GLU23 flipped	ASN106 flipped<br><br><br><br>GLU23 not flipped
JAK3	ASP967 is deprotonated and flipped	ASP967 is neutral
JNK2	HIS149 (NE2) is protonated and neutral, ND1 is not protonated & neutral<br><br>TH183 (HG1) is differently oriented.<br><br>HIS66 flipped	 <br><br><br><br>HIS66 flipped (HIE, NE2 is protonated and neutral)<br><br>HIS143 ND1 is protonated and charged (HIP)
MDM2	GLN59 (NE2) flipped near to ligand (no direct interaction)<br><br>HIS96 (ND1) flipped near to ligand (no direct interaction)<br><br><br><br>HIS73 (HIS, ND1 is protonated and neutral)	GLN59 (NE2) flipped near to ligand (no direct interaction)<br><br>HIS96 (ND1) flipped near to ligand (no direct interaction)<br><br>GLN71 flipped<br><br>HIS73 (HIE, NE2 is protonated and neutral)
P38-alpha	TYR35 (HH) is in different orientation<br><br>HIS107 (NE1) is only protonated and neutral<br><br>HIS185 (HIS) flipped	 <br><br><br>HIS185 (HIS) not flipped
PI3Kg	SER806 (HG) is differently oriented.<br><br>TYR867 (HH) is differently oriented.<br><br>THR887 (HG1) is differently	HI834 flipped

	oriented.<br><br>ASN949 flipped<br><br>HIS962 not flipped (HIE)	 <br>ASN949 flipped<br><br>HIS962 flipped (HIS)
PNP	HIS64 (NE2) is protonated and flipped near to ligand<br><br>HIS86 (NE2 & ND1) are protonated, (ND1) is charged, no flip<br><br>SER220 (OG) proton has different orientation	HIE64 flipped<br><br>HIP86 flipped (HIE and ND1 is protonated)
PPARg	HIS323 (flipped) (ND1) is protonated and charged, (NE2) is protonated and neutral and HB with ligand<br><br>TYR473 (HH) is differently oriented.	HIP323 (flipped) (ND1) is protonated and charged, (NE2) is protonated and neutral and HB with ligand<br><br><br><br>GLN273 flipped
THROMBIN	TYR60A (HH) is differently oriented.<br><br>SER195 (HG) is differently oriented.<br><br>SER214 (HG) is differently oriented.<br><br>TYR225 (HH) is differently oriented.	nothing special
TS	HIS196 (NE2) is protonated and neutral, the other N is neutral, HB with ligand<br><br>HIS261 HIP and NE2 is neutral while ND1 is protonated and charged<br><br><br><br>HIS 250 flipped<br><br>HIE196	 <br><br><br>HIS261<br><br>ASN260 flipped<br><br>HIS 250 flipped<br><br>HIS196

* The atom names are according to MOE conventional nomenclature.

Table S3. Overview of protoantaion/tautomerization states difference between MOE and Maestro schemes as well as the average number of the rotatable bonds, heavy atoms and pairwise RMSD per target.

Target	Num. of different protomers/tautomers per actives set	Num. of different protomers/tautomers per decoys set	Average num. of rotatable bonds per actives set	Average num. of rotatable bonds per dataset	Average num. of heavy atoms per actives set	Average num. of heavy atoms per dataset	Average pairwise RMSD per actives set	Average pairwise RMSD per dataset
ACE	2	435	8.6	8.3	27.8	27.6	2.5	2.3
ACHE	3	331	6.8	6.7	29.4	28.6	2.3	2.2
ADRB2	9	329	9.8	9.4	29.8	29.7	2.2	2.3
CATL	4	220	10.6	10.2	35.1	33.9	2.9	2.7
DHFR	20	345	5.5	5.4	26.9	26.8	1.8	1.8
ERBB2	27	260	8.6	8.3	34.7	33.8	2.1	2.1
HDAC2	29	275	7.5	7.3	28.1	27.5	1.7	1.8
HIVPR	6	184	12.2	11.4	45.4	41.2	4.4	3.6
HSP90	11	363	5.2	5.2	28.7	29.3	1.7	1.8
JAK3	14	341	4.5	4.5	29.7	29.0	2	1.9
JNK2	12	295	5.3	5.3	30.2	29.7	2.1	2
MDM2	13	256	7.3	7.2	37.4	36.4	2.5	2.4
P38	8	254	4.8	4.7	28.2	27.7	1.6	1.7
PI3KG	6	411	4	4.1	30.3	29.4	1.8	1.8
PNP	34	425	3.4	3.4	18.8	18.9	1.6	1.4
PPARg	5	266	9.4	9	33.0	32.6	2.8	2.5
Thrombin	9	350	8.8	8.6	34.3	33.5	2.7	2.5
TS	28	298	5.6	5.6	30.2	29.5	2.3	2.1

Figure S1. Scatter plot of the correlation between the percentage difference in protonation/tautomerization states of the bioactives and the absolute value of the ΔpROC-AUC.

(A)

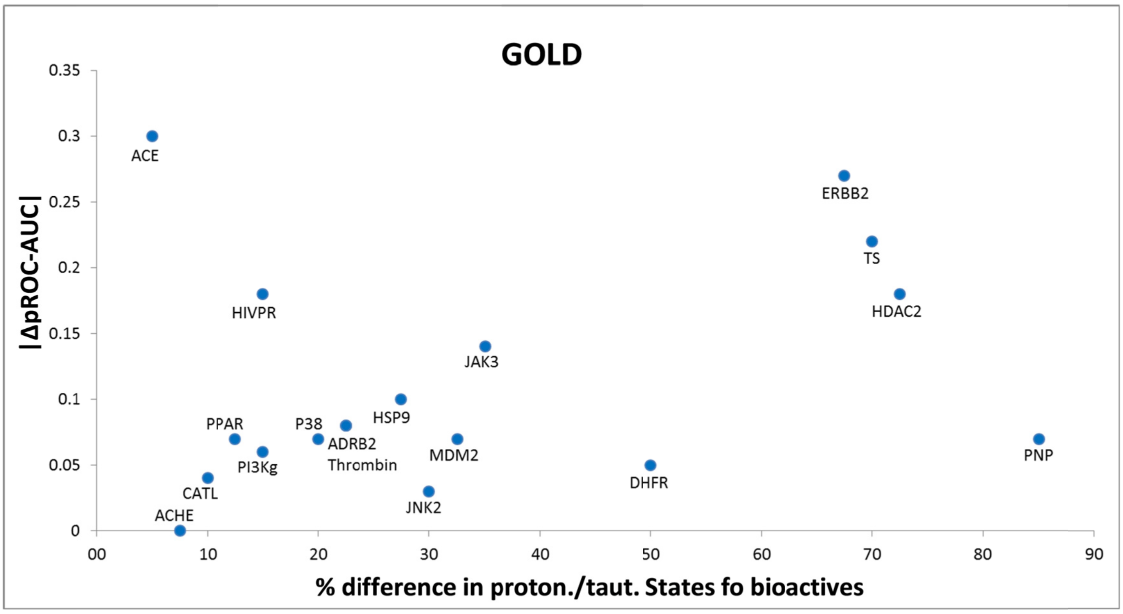

(B)

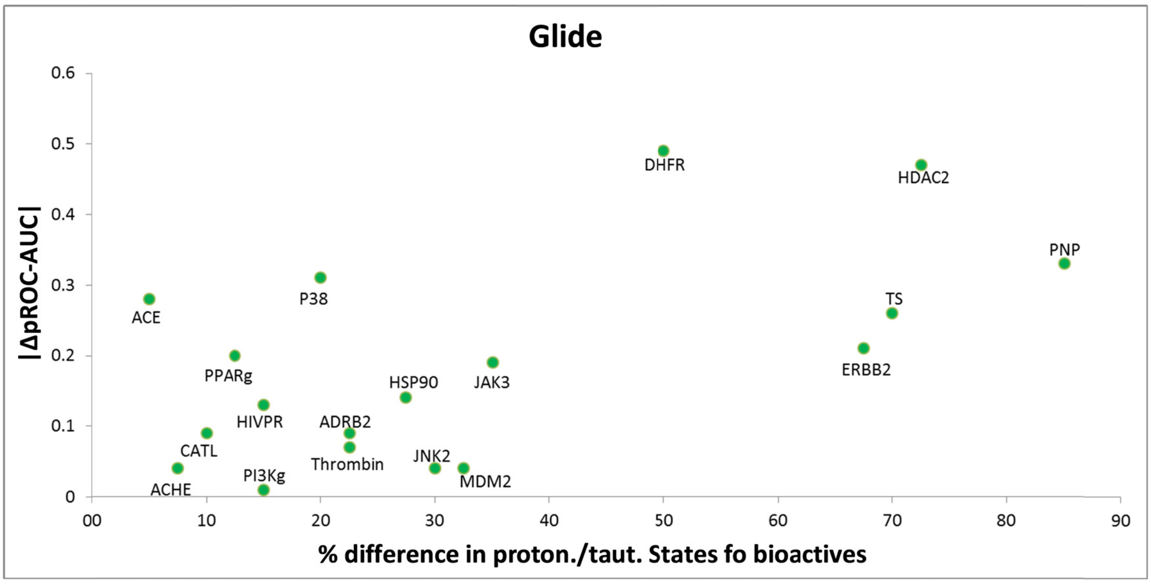

When ignoring the special cases of ACE (metal containing protein) and HIVPR (flexible binding site) for both (A) and (B), the R^2 value becomes 0.4 for both. This suggests that the correlation between difference in protonation/tautomerization states of the bioactives and ΔpROC-AUC$_{prep}$ shows certain trend.

Figure S2. Binding site of DHFR showing the difference between Maestro (A) and MOE (B). (C) Match vs mismatch assessment for DHFR benchmark sets using Glide.

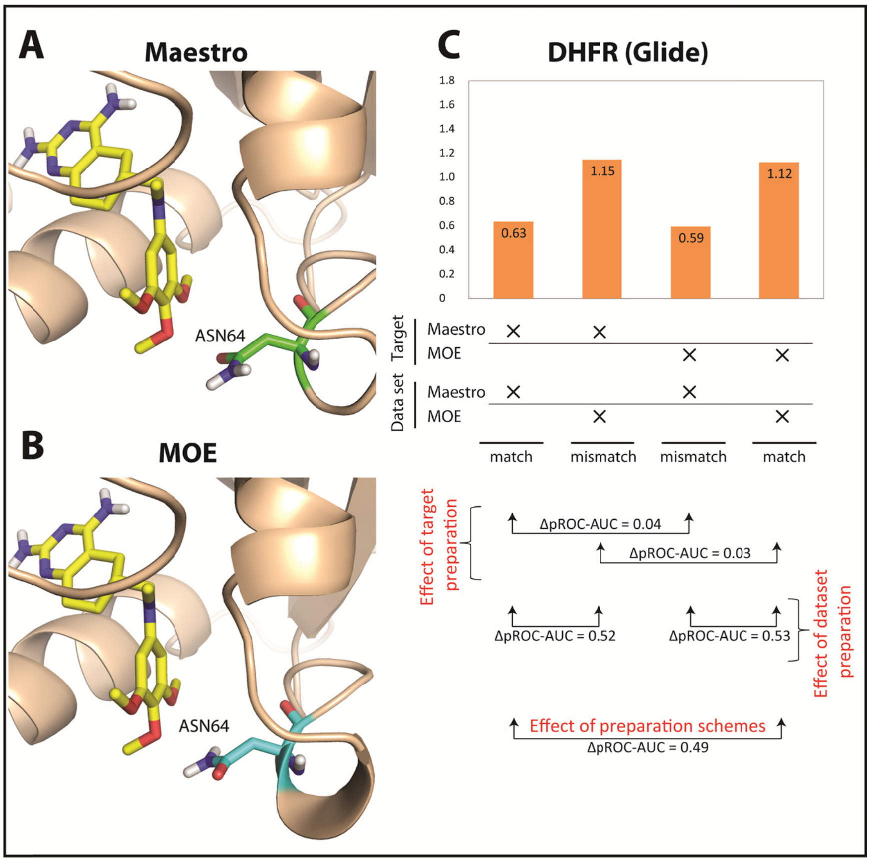

A and B show preparation difference between Maestro and MOE preparations since ASN-64 flipped 180° between both preparations. Match vs. mismatch assessment showed that the dataset preparation possesses the highest impact on the screening performance. Since the 20 bioactives out of 40 possess difference in protonation states between the two preparations (next figure, Figure S5), it is likely that the protonation selection of the bioactives set has the highest impact on the screening performance difference between the two preparations.

Figure S3. Overview of the protonation/tautomerization states selected by MOE and Maestro preparations for DHFR active set.

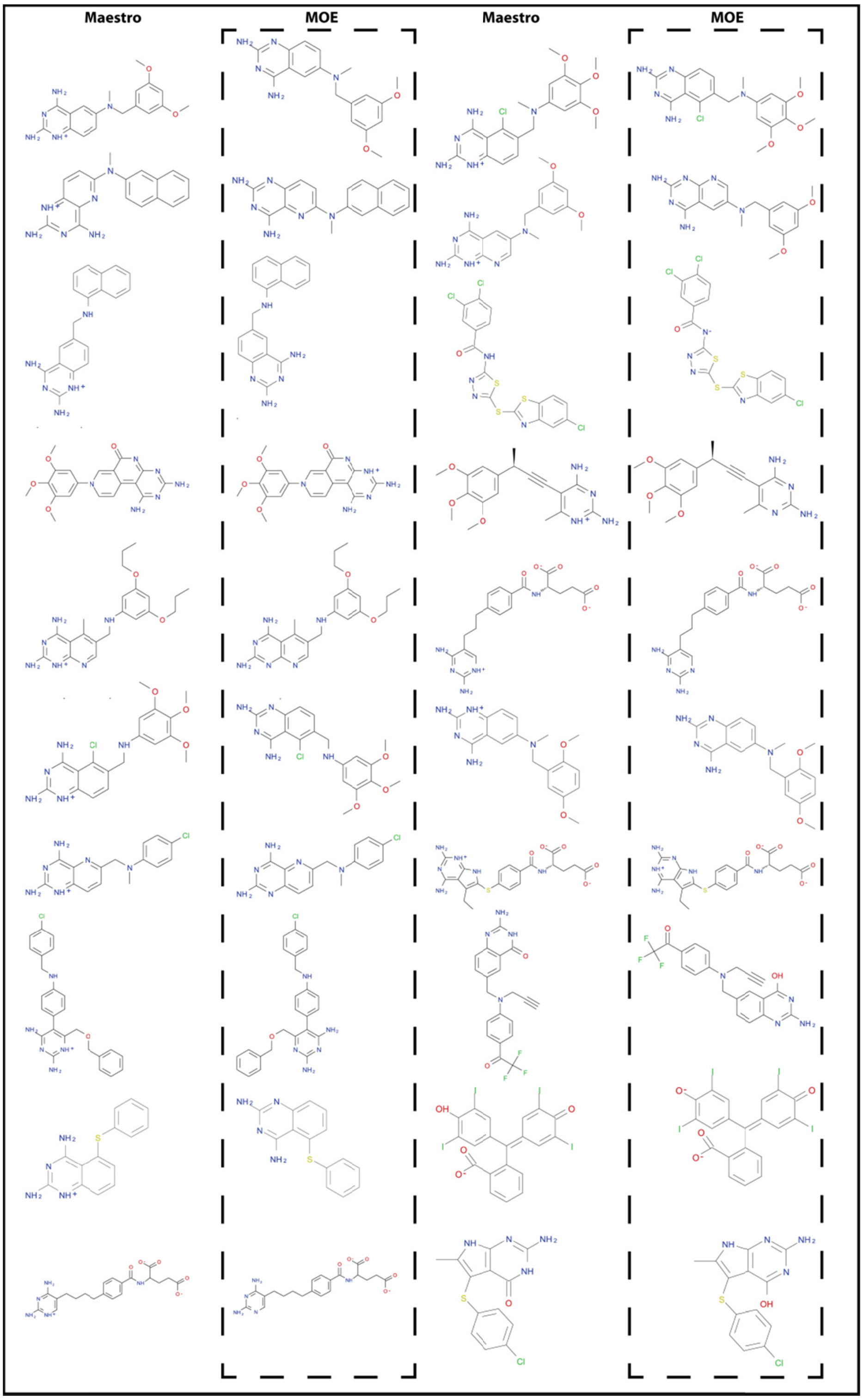

Figure S4. Overview of the protonation states selected by MOE and Maestro preparations for ACE bioactive set.

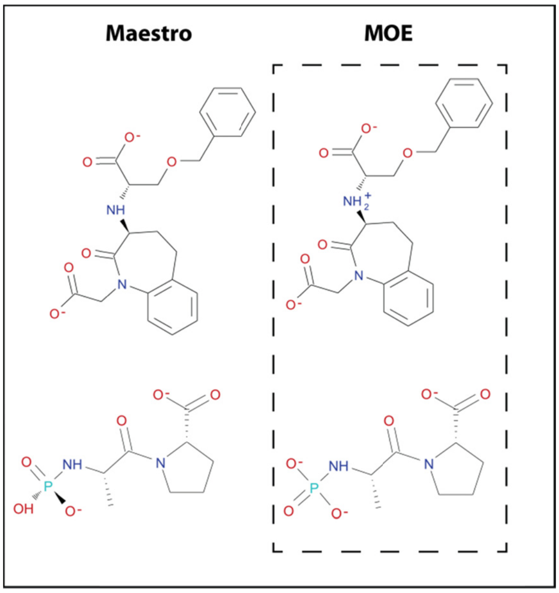

Figure S5. Scatter plot of the correlation between (Δ fitness) vs. (number of rotatable bonds). (A) When including the whole bioactives of ACE benchmark set. (B) When including cutoff > 7 number of rotatable bonds. Clearly no correlation was found.

(A)

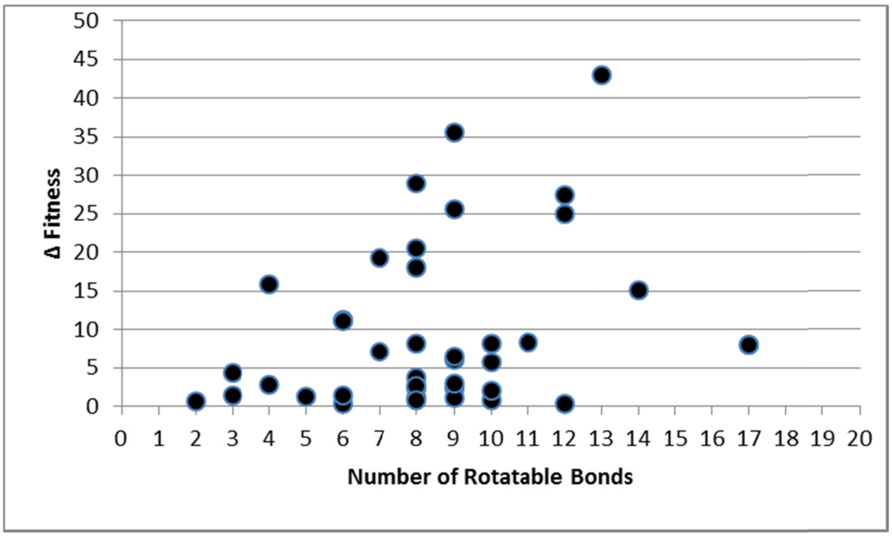

(B)

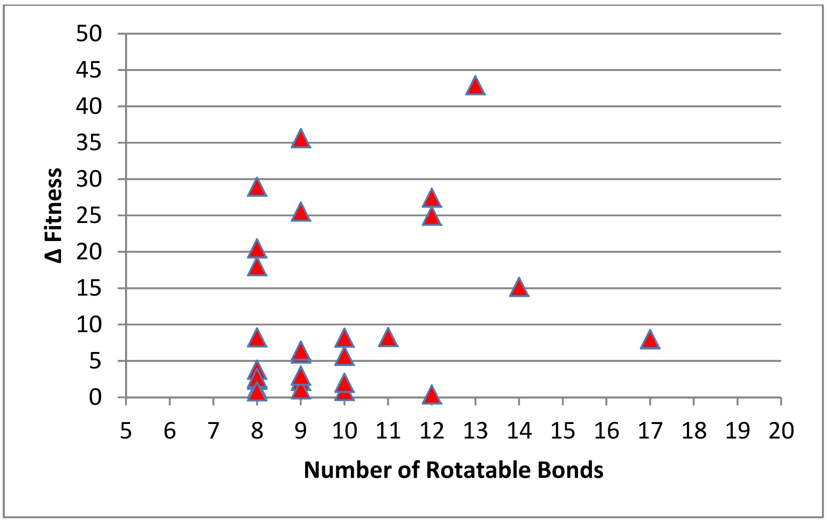

Figure S6. Scatter plot of the correlation between the number of heavy atoms and the pairwise RMSD of the two conformations resulted from Maestro and MOE preparations for ACE bioactives**.**

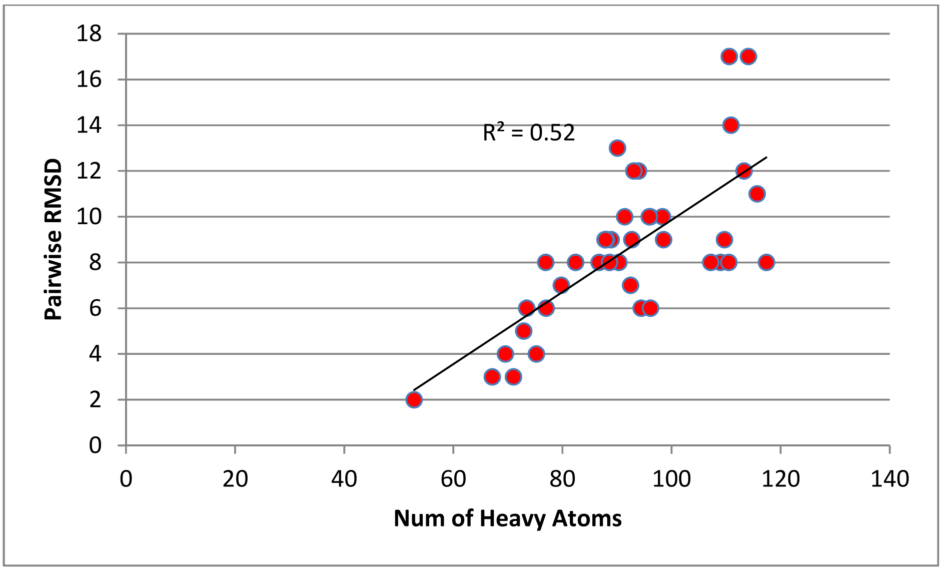

Table S4. Overview of the normalized performance of GOLD and Glide runs with the two preparations. Green color is for (ΔpROC AUCN > +0.05), and purple color is for (ΔpROC AUCN < -0.05). The original pROC-AUC values are in Table 1 in the manuscript.

Target	Glide								GOLD							
	MOE		Maestro		MOE		Maestro		MOE		Maestro		MOE		Maestro	
	$N^{2/3}$	ΔpROC-AUC$_N$[a]	$N^{2/3}$	ΔpROC-AUC$_N$	$N^{1/2}$	ΔpROC-AUC$_N$	$N^{1/2}$	ΔpROC-AUC$_N$	$N^{2/3}$	ΔpROC-AUC$_N$	$N^{2/3}$	ΔpROC-AUC$_N$	$N^{1/2}$	ΔpROC-AUC$_N$	$N^{1/2}$	ΔpROC-AUC$_N$
ACE	1.25	***-0.49***	1.46	***-0.55***	1.38	***-0.36***	1.64	***-0.37***	1.76	***0.21***	1.48	***0.23***	1.81	***0.26***	1.48	***0.23***
ACHE	0.60	***-0.11***	0.70	***-0.05***	0.65	***-0.06***	0.79	***0.03***	0.64	***-0.07***	0.64	***-0.08***	0.71	***-0.01***	0.71	***-0.01***
ADRB2	0.97	***-0.01***	0.84	***-0.05***	1.10	***0.13***	0.92	***0.03***	0.76	***0.02***	0.66	***0.01***	0.80	***0.07***	0.68	***0.03***
CATL	0.50	***-0.22***	0.59	***-0.22***	0.54	***-0.18***	0.64	***-0.17***	0.55	***-0.23***	0.54	***-0.20***	0.63	***-0.16***	0.61	***-0.14***
DHFR	0.95	***-0.17***	0.55	***-0.08***	1.06	***-0.06***	0.60	***-0.03***	1.07	***0.32***	1.05	***0.25***	1.02	***0.27***	1.05	***0.25***
ERBB2	1.30	***-0.71***	1.04	***-0.76***	1.59	***-0.42***	1.24	***-0.56***	1.00	***-0.85***	1.53	***-0.59***	1.34	***-0.51***	1.85	***-0.27***
HDAC2	1.36	***-0.38***	1.01	***-0.26***	1.46	***-0.28***	1.07	***-0.20***	0.93	***-0.09***	1.30	***0.10***	1.03	***0.00***	1.46	***0.26***
HIVPR	1.00	***-0.54***	0.94	***-0.46***	1.22	***-0.32***	1.09	***-0.31***	0.75	***-0.70***	0.86	***-0.78***	0.97	***-0.48***	1.14	***-0.50***
HSP90	0.68	***-0.06***	0.59	***-0.01***	0.73	***-0.01***	0.61	***0.00***	0.58	***0.16***	0.46	***0.14***	0.55	***0.12***	0.42	***0.10***
JAK3	0.97	***-0.36***	0.84	***-0.30***	1.06	***-0.27***	0.92	***-0.21***	0.76	***-0.02***	0.90	***-0.01***	0.82	***0.04***	0.99	***0.07***
JNK2	0.51	***-0.14***	0.54	***-0.16***	0.60	***-0.06***	0.62	***-0.08***	0.68	***-0.09***	0.64	***-0.10***	0.71	***-0.05***	0.69	***-0.05***
MDM2	0.63	***-0.22***	0.60	***-0.21***	0.67	***-0.17***	0.64	***-0.16***	0.35	***-0.16***	0.29	***-0.15***	0.40	***-0.11***	0.34	***-0.11***
P38	1.23	***-0.56***	1.04	***-0.44***	1.44	***-0.36***	1.18	***-0.30***	0.53	***0.04***	0.59	***0.03***	0.53	***0.04***	0.60	***0.04***

PI3KG	0.78	***-0.36***	0.80	***-0.33***	0.86	***-0.28***	0.87	***-0.26***	1.02	***0.02***	0.98	***0.03***	1.17	***0.16***	1.10	***0.15***
PNP	0.95	***-0.13***	1.17	***-0.24***	1.06	***-0.02***	1.33	***-0.08***	1.22	***0.24***	1.37	***0.32***	1.24	***0.26***	1.38	***0.34***
PPARg	0.73	***-0.17***	0.85	***-0.26***	0.85	***-0.05***	1.00	***-0.11***	0.83	***-0.02***	0.84	***-0.08***	0.89	***0.04***	0.93	***0.01***
Thrombin	1.61	***-0.39***	1.51	***-0.42***	1.76	***-0.24***	1.62	***-0.31***	1.12	***-0.11***	1.03	***-0.12***	1.20	***-0.03***	1.09	***-0.06***
TS	0.64	***-0.58***	0.70	***-0.77***	0.73	***-0.49***	0.82	***-0.66***	1.06	***-0.21***	0.85	***-0.20***	1.26	***-0.01***	1.06	***0.01***

[a]ΔpROC-AUC$_N$ is calculated as (pROC-AUC of normalized – pROC-AUC of original).
